# 2-[(*E*)-2-(4-Chloro­phen­yl)ethen­yl]-1-methyl­pyridinium 4-methoxy­benzene­sulfonate

**DOI:** 10.1107/S1600536809021679

**Published:** 2009-06-10

**Authors:** Kullapa Chanawanno, Suchada Chantrapromma, Hoong-Kun Fun

**Affiliations:** a Crystal Materials Research Unit, Department of Chemistry, Faculty of Science, Prince of Songkla University, Hat-Yai, Songkhla 90112, Thailand; b X-ray Crystallography Unit, School of Physics, Universiti Sains Malaysia, 11800 USM, Penang, Malaysia

## Abstract

In the asymmetric unit of the title salt, C_14_H_13_ClN^+^·C_7_H_7_O_4_S^−^, there are two crystallographically independent mol­ecules for each component. Each cation adopts an *E* configuration with respect to the C=C bond and is slightly twisted; the dihedral angle between the pyridinium and benzene rings is 6.53 (7)° for one mol­ecule and 5.30 (7)° for the other. The meth­oxy groups in the anion mol­ecules are each twisted from the mean plane of benzene ring with torsion angles of 16.38 (19) and 4.32 (19)°. In the crystal structure, the cations are stacked in an anti­parallel manner along the *a* axis and the anions are linked together by C—H⋯O inter­actions into a layer parallel to (001). The anion layers are further linked to adjacent cations by C—H⋯O inter­actions. C—H⋯π inter­actions involving the benzene rings of both ions are also observed.

## Related literature

For bond length data, see: Allen *et al.* (1987 ▶). For background on non-linear optical materials research, see: Cheng, Tam, Marder *et al.* (1991 ▶); Cheng, Tam, Stevenson *et al.* (1991 ▶). For related structures, see: Chanawanno *et al.* (2008 ▶); Chantrapromma *et al.* (2009 ▶); Chantrapromma, Rodwatcharapiban & Fun (2006 ▶); Chantrapromma, Ruanwas *et al.* (2006 ▶). For the stability of the temperature controller used in the data collection, see: Cosier & Glazer (1986 ▶).
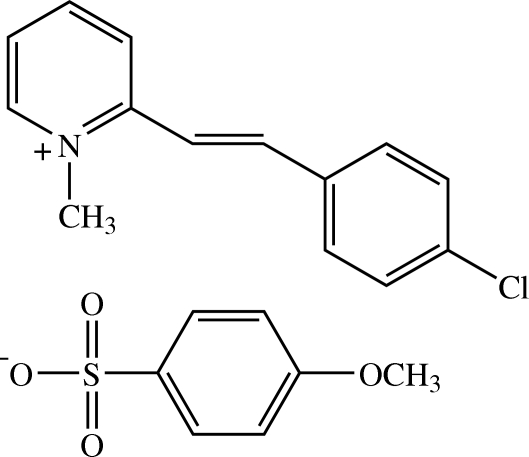


## Experimental

### 

#### Crystal data

C_14_H_13_ClN^+^·C_7_H_7_O_4_S^−^
*M* *_r_* = 417.90
Monoclinic, 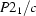

*a* = 15.2323 (2) Å
*b* = 14.2222 (2) Å
*c* = 17.9709 (3) Å
β = 99.842 (1)°
*V* = 3835.86 (10) Å^3^
*Z* = 8
Mo *K*α radiation
μ = 0.34 mm^−1^
*T* = 100 K
0.50 × 0.31 × 0.25 mm

#### Data collection

Bruker APEXII CCD area-detector diffractometer
Absorption correction: multi-scan (**SADABS**; Bruker, 2005 ▶) *T*_min_ = 0.849, *T*_max_ = 0.921
65482 measured reflections
13850 independent reflections
11056 reflections with *I* > 2σ(*I*)
*R*_int_ = 0.033

#### Refinement

*R*[*F* ^2^ > 2σ(*F* ^2^)] = 0.043
*wR*(*F* ^2^) = 0.124
*S* = 1.05
13850 reflections
509 parameters
H-atom parameters constrained
Δρ_max_ = 0.97 e Å^−3^
Δρ_min_ = −0.36 e Å^−3^

### 

Data collection: *APEX2* (Bruker, 2005 ▶); cell refinement: *SAINT* (Bruker, 2005 ▶); data reduction: *SAINT*; program(s) used to solve structure: *SHELXTL* (Sheldrick, 2008 ▶); program(s) used to refine structure: *SHELXTL*; molecular graphics: *SHELXTL*; software used to prepare material for publication: *SHELXTL* and *PLATON* (Spek, 2009 ▶).

## Supplementary Material

Crystal structure: contains datablocks global, I. DOI: 10.1107/S1600536809021679/is2428sup1.cif

Structure factors: contains datablocks I. DOI: 10.1107/S1600536809021679/is2428Isup2.hkl

Additional supplementary materials:  crystallographic information; 3D view; checkCIF report

## Figures and Tables

**Table 1:** Hydrogen-bond geometry (Å, °)

*D*—H⋯*A*	*D*—H	H⋯*A*	*D*⋯*A*	*D*—H⋯*A*
C1*A*—H1*AA*⋯O3*B*^i^	0.93	2.53	3.3040 (17)	141
C7*A*—H7*AB*⋯O3*A*^ii^	0.96	2.56	3.506 (2)	171
C1*B*—H1*BA*⋯O3*A*	0.93	2.31	3.2373 (17)	172
C12*B*—H12*B*⋯O2*B*^i^	0.93	2.47	3.2817 (17)	146
C7*B*—H7*BA*⋯O4*A*^ii^	0.96	2.57	3.518 (2)	171
C14*B*—H14*B*⋯O3*B*^i^	0.93	2.59	3.5204 (19)	176
C15*A*—H15*A*⋯O2*A*^iii^	0.93	2.47	3.2626 (19)	143
C17*B*—H17*B*⋯O3*B*^i^	0.93	2.58	3.474 (2)	161
C20*A*—H20*A*⋯O4*A*^iv^	0.93	2.50	3.159 (2)	128
C20*B*—H20*B*⋯O4*B*^v^	0.93	2.38	3.185 (2)	144
C21*A*—H21*C*⋯O2*A*^iii^	0.96	2.29	3.162 (2)	150
C21*B*—H21*D*⋯O2*B*^vi^	0.96	2.42	3.317 (2)	156
C9*A*—H9*AA*⋯*Cg*3^iii^	0.93	2.70	3.4423 (13)	137
C11*A*—H11*A*⋯*Cg*3	0.93	2.59	3.3482 (13)	139
C11*B*—H11*B*⋯*Cg*4^vii^	0.93	2.77	3.5457 (14)	142
C9*B*—H9*BA*⋯*Cg*4^vi^	0.93	2.62	3.3442 (13)	135
C21*A*—H21*B*⋯*Cg*2^iii^	0.96	2.82	3.6853 (18)	151
C21*B*—H21*F*⋯*Cg*1^viii^	0.96	2.82	3.7100 (19)	155

Symmetry codes: (i) 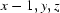; (ii) 
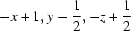
; (iii) 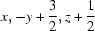; (iv) 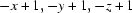; (v) 
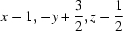
; (vi) 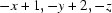; (vii) 
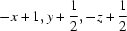
; (viii) 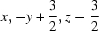. *Cg*1, *Cg*2, *Cg*3 and *Cg*4 are the centroids of the C8*A*–C13*A*, C8*B*–C13*B*, C1*A*–C6*A* and C1*B*–C6*B* rings, respectively.

**Fig. 1.**
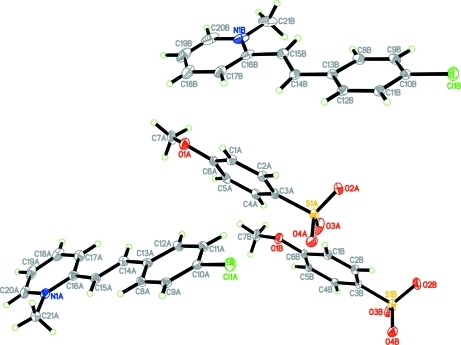
The molecular structure of the title compound, with 40% probability displacement ellipsoids and the atom-numbering scheme.

**Fig. 2.**
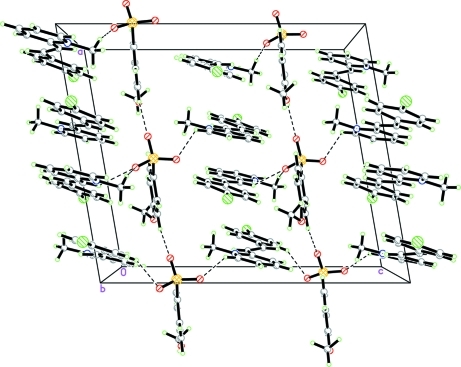
The crystal packing of the title compound viewed down the b axis. Weak C—H···O interactions are shown as dashed lines.

## supplementary crystallographic information

### Comment

In search for new organic nonlinear optical (NLO) materials, aromatic compounds with donor and acceptor substituents are extensively studied. Styryl pyridinium derivatives are considered to be promising NLO materials (Cheng, Tam, Marder *et al.*, 1991; Cheng, Tam, Stevenson *et al.*, 1991). During the course of our exploring for new organic NLO materials, we have previously synthesized and reported a number of the crystal structures of pyridinium derivatives (Chanawanno *et al.*, 2008; Chantrapromma, Rodwatcharapiban & Fun, 2006; Chantrapromma, Ruanwas *et al.*, 2006; Chantrapromma *et al.*, 2009). The title compound (I) has been synthesized and its crystal structure is reported here as part of our ongoing research on NLO materials. Unfortunately, the title compound crystallized in centrosymmetric space group *P*2_1_/*c* and does not exhibit second-order nonlinear optical properties.

The asymmetric unit of (I) contains  two crystallographically independent molecules each of the C_14_H_13_ClN^+^ cation and the C_7_H_7_O_4_S^-^ anion which are almost identical (Fig. 1). The cation adopts the *E* configuration with respect to the C14═C15 double bond [1.3429 (19) Å in molecule *A* and 1.3445 (19) Å in molecule *B*] and the torsion angle of C13–C14–C15–C16 = 178.86 (12)° in molecule *A* [178.33 (12)° in molecule *B*]. The molecule of cation is slightly twisted which can be indicated by the dihedral angle between the pyridinium and benzene rings of the cation being 6.53 (7)° in molecule *A* [5.30 (7)° in molecule *B*]. The methoxy group is twisted from the plane of benzene ring of the anion with the torsion angle C7–O1–C6–C5 = -16.38 (19)° in molecule *A* [-4.32 (19)° in molecule *B*]. The bond distances in both cation and anion have normal values (Allen *et al.*, 1987) and comparable with the closely related compounds (Chanawanno *et al.*, 2008; Chantrapromma, Rodwatcharapiban & Fun, 2006; Chantrapromma, Ruanwas *et al.*, 2006; Chantrapromma *et al.*, 2009).

In the crystal packing (Fig. 2), all O atoms of the sulfonate group are involved in weak C—H···O interactions (Table 1). The cations and anions are alternately arranged with the cations stacked in an antiparallel manner along the *a* axis and the anions linked together by C—H···O weak interactions (Table 1) into chains along the same direction. The anion chains are further linked to the adjacent cations into ribbons along the *c* axis by C—H···O weak interactions (Table 1). The crystal structure is further stabilized by C—H···π interactions involving the benzene rings (Table 1); *Cg*1, *Cg*2, *Cg*3 and *Cg*4 are the centroids of the C8A–C13A, C8B–C13B, C1A–C6A and C1B–C6B rings, respectively.

### Experimental

2-[(*E*)-2-(4-chlorophenyl)ethenyl]-1-methylpyridinium iodide (0.24 g, 0.67 mmol) was prepared according to the previous report (Chanawanno *et al.*, 2008) and mixed with silver(I) 4-methoxybenzenesulfonate (0.19 g, 0.67 mmol) (Chantrapromma, Rodwatcharapiban & Fun, 2006) in methanol solution (100 ml). The mixture solution was stirred for 30 min, the precipitate of silver iodide which formed was filtered and the filtrate was evaporated to give the title compound as an orange solid. Orange block-shaped single crystals of the title compound suitable for X-ray structure determination were recrystallized from methanol by slow evaporation at room temperature over a few weeks (m.p. 476–477 K).

### Refinement

All H atoms were positioned geometrically and allowed to ride on their parent atoms, with d(C—H) = 0.93 Å for aromatic and CH, and 0.96 Å for CH_3_. The *U*_iso_ values were constrained to be 1.5*U*_eq_ of the carrier atom for methyl H atoms and 1.2*U*_eq_ for the remaining H atoms. A rotating group model was used for the methyl groups. The highest residual electron density peak is located at 0.79 Å from Cl1A and the deepest hole is located at 0.61 Å from S1A.

### Crystal data

**Table:**

C_14_H_13_ClN^+^·C_7_H_7_O_4_S^−^	*F*(000) = 1744
*M**_r_* = 417.90	*D*_x_ = 1.447 Mg m^−^^3^
Monoclinic, *P*2_1_/*c*	Melting point = 476–477 K
Hall symbol: -P 2ybc	Mo *K*α radiation, λ = 0.71073 Å
*a* = 15.2323 (2) Å	Cell parameters from 13850 reflections
*b* = 14.2222 (2) Å	θ = 2.2–32.5°
*c* = 17.9709 (3) Å	µ = 0.34 mm^−^^1^
β = 99.842 (1)°	*T* = 100 K
*V* = 3835.86 (10) Å^3^	Block, orange
*Z* = 8	0.50 × 0.31 × 0.25 mm

### Data collection

**Table:**

Bruker APEXII CCD area-detector diffractometer	13850 independent reflections
Radiation source: sealed tube	11056 reflections with *I* > 2σ(*I*)
graphite	*R*_int_ = 0.033
φ and ω scans	θ_max_ = 32.5°, θ_min_ = 2.2°
Absorption correction: multi-scan (*SADABS*; Bruker, 2005)	*h* = −22→22
*T*_min_ = 0.849, *T*_max_ = 0.921	*k* = −21→19
65482 measured reflections	*l* = −27→25

### Refinement

**Table:**

Refinement on *F*^2^	Primary atom site location: structure-invariant direct methods
Least-squares matrix: full	Secondary atom site location: difference Fourier map
*R*[*F*^2^ > 2σ(*F*^2^)] = 0.043	Hydrogen site location: inferred from neighbouring sites
*wR*(*F*^2^) = 0.124	H-atom parameters constrained
*S* = 1.05	*w* = 1/[σ^2^(*F*_o_^2^) + (0.0623*P*)^2^ + 1.6315*P*] where *P* = (*F*_o_^2^ + 2*F*_c_^2^)/3
13850 reflections	(Δ/σ)_max_ = 0.001
509 parameters	Δρ_max_ = 0.97 e Å^−^^3^
0 restraints	Δρ_min_ = −0.36 e Å^−^^3^

### Special details

**Table:**

Experimental. The crystal was placed in the cold stream of an Oxford Cryosystems Cobra open-flow nitrogen cryostat (Cosier & Glazer, 1986) operating at 100.0 (1) K.
Geometry. All esds (except the esd in the dihedral angle between two l.s. planes) are estimated using the full covariance matrix. The cell esds are taken into account individually in the estimation of esds in distances, angles and torsion angles; correlations between esds in cell parameters are only used when they are defined by crystal symmetry. An approximate (isotropic) treatment of cell esds is used for estimating esds involving l.s. planes.
Refinement. Refinement of F^2^ against ALL reflections. The weighted R-factor wR and goodness of fit S are based on F^2^, conventional R-factors R are based on F, with F set to zero for negative F^2^. The threshold expression of F^2^ > 2sigma(F^2^) is used only for calculating R-factors(gt) etc. and is not relevant to the choice of reflections for refinement. R-factors based on F^2^ are statistically about twice as large as those based on F, and R- factors based on ALL data will be even larger.

### Fractional atomic coordinates and isotropic or equivalent isotropic displacement parameters (Å^2^)

**Table:**

	*x*	*y*	*z*	*U*_iso_*/*U*_eq_	
Cl1A	0.32287 (2)	0.88225 (2)	0.45723 (2)	0.02793 (8)	
S1A	0.48109 (2)	0.93858 (2)	0.237547 (19)	0.01944 (7)	
O1A	0.23458 (7)	0.61426 (7)	0.18741 (6)	0.0284 (2)	
O2A	0.44566 (8)	1.00518 (8)	0.17884 (7)	0.0341 (3)	
O3A	0.56868 (7)	0.90330 (9)	0.23050 (9)	0.0404 (3)	
O4A	0.47682 (7)	0.97356 (8)	0.31282 (6)	0.0275 (2)	
N1A	0.39490 (7)	0.25104 (9)	0.56571 (7)	0.0238 (2)	
C1A	0.26165 (8)	0.77407 (10)	0.21133 (7)	0.0209 (2)	
H1AA	0.2012	0.7817	0.2120	0.025*	
C2A	0.31827 (8)	0.85064 (9)	0.22216 (7)	0.0190 (2)	
H2AA	0.2954	0.9100	0.2291	0.023*	
C3A	0.40971 (8)	0.83949 (9)	0.22282 (7)	0.0174 (2)	
C4A	0.44290 (9)	0.75035 (9)	0.21200 (7)	0.0193 (2)	
H4AA	0.5037	0.7425	0.2129	0.023*	
C5A	0.38663 (9)	0.67272 (9)	0.19984 (7)	0.0211 (2)	
H5AA	0.4094	0.6135	0.1921	0.025*	
C6A	0.29572 (9)	0.68487 (9)	0.19935 (7)	0.0205 (2)	
C7A	0.26202 (13)	0.52937 (11)	0.15488 (9)	0.0347 (4)	
H7AA	0.2114	0.4890	0.1408	0.052*	
H7AB	0.3058	0.4979	0.1911	0.052*	
H7AC	0.2872	0.5444	0.1109	0.052*	
C8A	0.34428 (9)	0.61220 (10)	0.51834 (7)	0.0206 (2)	
H8AA	0.3340	0.5758	0.5590	0.025*	
C9A	0.32870 (9)	0.70814 (10)	0.51876 (7)	0.0212 (2)	
H9AA	0.3075	0.7360	0.5590	0.025*	
C10A	0.34515 (8)	0.76217 (9)	0.45819 (7)	0.0202 (2)	
C11A	0.37708 (9)	0.72212 (10)	0.39728 (7)	0.0214 (2)	
H11A	0.3886	0.7593	0.3575	0.026*	
C12A	0.39139 (9)	0.62607 (10)	0.39716 (7)	0.0210 (2)	
H12A	0.4120	0.5987	0.3564	0.025*	
C13A	0.37542 (8)	0.56894 (9)	0.45724 (7)	0.0187 (2)	
C14A	0.39199 (8)	0.46781 (10)	0.45331 (8)	0.0207 (2)	
H14A	0.4060	0.4441	0.4086	0.025*	
C15A	0.38828 (9)	0.40694 (10)	0.50982 (8)	0.0217 (2)	
H15A	0.3753	0.4305	0.5550	0.026*	
C16A	0.40336 (8)	0.30628 (10)	0.50458 (8)	0.0215 (2)	
C17A	0.42550 (9)	0.26255 (11)	0.44091 (9)	0.0264 (3)	
H17A	0.4318	0.2986	0.3989	0.032*	
C18A	0.43828 (10)	0.16620 (11)	0.43922 (11)	0.0317 (3)	
H18A	0.4520	0.1374	0.3962	0.038*	
C19A	0.43033 (10)	0.11325 (11)	0.50268 (11)	0.0342 (4)	
H19A	0.4394	0.0486	0.5028	0.041*	
C20A	0.40912 (10)	0.15698 (11)	0.56505 (10)	0.0307 (3)	
H20A	0.4044	0.1216	0.6077	0.037*	
C21A	0.36931 (11)	0.29182 (11)	0.63493 (9)	0.0294 (3)	
H21A	0.3692	0.2433	0.6720	0.044*	
H21B	0.3108	0.3188	0.6228	0.044*	
H21C	0.4113	0.3397	0.6547	0.044*	
Cl1B	0.17518 (2)	1.38107 (3)	0.00463 (2)	0.02803 (8)	
S1B	1.00942 (2)	0.95490 (2)	0.246406 (19)	0.01974 (7)	
O1B	0.71704 (7)	0.67561 (7)	0.20958 (6)	0.0260 (2)	
O2B	0.98081 (7)	1.02649 (8)	0.18966 (7)	0.0305 (2)	
O3B	1.09004 (7)	0.90609 (8)	0.23490 (8)	0.0344 (3)	
O4B	1.01406 (8)	0.98951 (8)	0.32302 (6)	0.0303 (2)	
N1B	0.10047 (8)	0.74523 (10)	−0.05082 (8)	0.0319 (3)	
C1B	0.76794 (8)	0.83076 (9)	0.22112 (7)	0.0200 (2)	
H1BA	0.7091	0.8498	0.2189	0.024*	
C2B	0.83556 (8)	0.89695 (9)	0.22830 (7)	0.0191 (2)	
H2BA	0.8219	0.9606	0.2298	0.023*	
C3B	0.92421 (8)	0.86844 (9)	0.23329 (7)	0.0171 (2)	
C4B	0.94376 (8)	0.77349 (9)	0.22876 (7)	0.0186 (2)	
H4BA	1.0027	0.7546	0.2316	0.022*	
C5B	0.87619 (9)	0.70594 (9)	0.22008 (7)	0.0189 (2)	
H5BA	0.8896	0.6425	0.2163	0.023*	
C6B	0.78849 (8)	0.73524 (9)	0.21724 (7)	0.0190 (2)	
C7B	0.73447 (11)	0.57794 (11)	0.19885 (11)	0.0347 (4)	
H7BA	0.6797	0.5432	0.1939	0.052*	
H7BB	0.7754	0.5549	0.2416	0.052*	
H7BC	0.7600	0.5703	0.1539	0.052*	
C8B	0.15924 (9)	1.10411 (10)	−0.02634 (7)	0.0228 (3)	
H8BA	0.1699	1.0617	−0.0632	0.027*	
C9B	0.17369 (9)	1.19907 (10)	−0.03607 (7)	0.0230 (3)	
H9BA	0.1943	1.2203	−0.0789	0.028*	
C10B	0.15711 (8)	1.26222 (10)	0.01867 (7)	0.0202 (2)	
C11B	0.12748 (9)	1.23201 (10)	0.08390 (7)	0.0211 (2)	
H11B	0.1170	1.2750	0.1204	0.025*	
C12B	0.11402 (8)	1.13654 (10)	0.09326 (7)	0.0203 (2)	
H12B	0.0948	1.1156	0.1368	0.024*	
C13B	0.12876 (8)	1.07074 (9)	0.03831 (7)	0.0189 (2)	
C14B	0.11282 (8)	0.97080 (10)	0.05008 (7)	0.0207 (2)	
H14B	0.1086	0.9508	0.0986	0.025*	
C15B	0.10399 (9)	0.90638 (10)	−0.00546 (8)	0.0234 (3)	
H15B	0.1067	0.9267	−0.0542	0.028*	
C16B	0.09044 (9)	0.80646 (10)	0.00640 (9)	0.0248 (3)	
C17B	0.06762 (9)	0.76977 (10)	0.07236 (9)	0.0273 (3)	
H17B	0.0596	0.8104	0.1112	0.033*	
C18B	0.05662 (10)	0.67402 (11)	0.08135 (12)	0.0353 (4)	
H18B	0.0423	0.6501	0.1260	0.042*	
C19B	0.06737 (11)	0.61438 (12)	0.02235 (13)	0.0424 (5)	
H19B	0.0605	0.5498	0.0272	0.051*	
C20B	0.08801 (11)	0.65085 (13)	−0.04276 (12)	0.0426 (5)	
H20B	0.0937	0.6106	−0.0825	0.051*	
C21B	0.12482 (12)	0.77952 (14)	−0.12228 (9)	0.0411 (4)	
H21D	0.0800	0.8224	−0.1462	0.062*	
H21E	0.1290	0.7272	−0.1553	0.062*	
H21F	0.1812	0.8112	−0.1118	0.062*	

### Atomic displacement parameters (Å^2^)

**Table:**

	*U*^11^	*U*^22^	*U*^33^	*U*^12^	*U*^13^	*U*^23^
Cl1A	0.03154 (17)	0.01948 (15)	0.03326 (18)	0.00150 (12)	0.00692 (13)	−0.00094 (13)
S1A	0.01734 (13)	0.01703 (14)	0.02512 (15)	0.00031 (10)	0.00697 (11)	0.00271 (11)
O1A	0.0329 (5)	0.0219 (5)	0.0302 (5)	−0.0082 (4)	0.0051 (4)	−0.0025 (4)
O2A	0.0464 (7)	0.0225 (5)	0.0301 (6)	−0.0095 (5)	−0.0029 (5)	0.0093 (4)
O3A	0.0186 (5)	0.0319 (6)	0.0749 (9)	−0.0011 (4)	0.0198 (5)	−0.0077 (6)
O4A	0.0284 (5)	0.0306 (6)	0.0236 (5)	−0.0073 (4)	0.0045 (4)	−0.0025 (4)
N1A	0.0178 (5)	0.0243 (6)	0.0276 (6)	−0.0037 (4)	−0.0006 (4)	0.0044 (5)
C1A	0.0183 (5)	0.0230 (6)	0.0217 (6)	0.0006 (4)	0.0045 (4)	−0.0004 (5)
C2A	0.0186 (5)	0.0172 (5)	0.0218 (6)	0.0040 (4)	0.0052 (4)	0.0001 (5)
C3A	0.0187 (5)	0.0144 (5)	0.0202 (5)	0.0023 (4)	0.0066 (4)	0.0025 (4)
C4A	0.0219 (5)	0.0172 (6)	0.0203 (6)	0.0053 (4)	0.0078 (4)	0.0035 (4)
C5A	0.0283 (6)	0.0158 (6)	0.0205 (6)	0.0042 (4)	0.0077 (5)	0.0026 (5)
C6A	0.0262 (6)	0.0183 (6)	0.0174 (5)	−0.0027 (4)	0.0044 (4)	0.0013 (4)
C7A	0.0586 (11)	0.0199 (7)	0.0273 (7)	−0.0119 (7)	0.0122 (7)	−0.0014 (6)
C8A	0.0213 (5)	0.0239 (6)	0.0171 (5)	−0.0003 (4)	0.0047 (4)	0.0012 (5)
C9A	0.0228 (6)	0.0238 (6)	0.0170 (5)	−0.0005 (5)	0.0036 (4)	−0.0025 (5)
C10A	0.0194 (5)	0.0192 (6)	0.0209 (6)	−0.0007 (4)	0.0007 (4)	−0.0012 (5)
C11A	0.0207 (5)	0.0253 (6)	0.0182 (6)	−0.0007 (5)	0.0036 (4)	0.0028 (5)
C12A	0.0212 (6)	0.0245 (6)	0.0181 (5)	0.0029 (5)	0.0059 (4)	0.0017 (5)
C13A	0.0166 (5)	0.0211 (6)	0.0182 (5)	0.0011 (4)	0.0027 (4)	−0.0003 (4)
C14A	0.0199 (5)	0.0214 (6)	0.0213 (6)	0.0010 (4)	0.0044 (4)	−0.0005 (5)
C15A	0.0227 (6)	0.0200 (6)	0.0218 (6)	−0.0002 (4)	0.0022 (5)	−0.0003 (5)
C16A	0.0164 (5)	0.0208 (6)	0.0261 (6)	−0.0006 (4)	0.0008 (4)	0.0026 (5)
C17A	0.0230 (6)	0.0253 (7)	0.0311 (7)	0.0017 (5)	0.0056 (5)	−0.0006 (6)
C18A	0.0221 (6)	0.0251 (7)	0.0480 (9)	0.0013 (5)	0.0062 (6)	−0.0055 (7)
C19A	0.0225 (6)	0.0199 (7)	0.0596 (11)	−0.0001 (5)	0.0053 (7)	0.0023 (7)
C20A	0.0217 (6)	0.0231 (7)	0.0451 (9)	−0.0039 (5)	−0.0003 (6)	0.0103 (6)
C21A	0.0321 (7)	0.0303 (8)	0.0249 (7)	−0.0098 (6)	0.0025 (5)	0.0035 (6)
Cl1B	0.02942 (16)	0.02355 (16)	0.03146 (18)	−0.00345 (12)	0.00619 (13)	0.00262 (13)
S1B	0.01986 (14)	0.01587 (14)	0.02444 (15)	−0.00113 (10)	0.00647 (11)	0.00002 (11)
O1B	0.0241 (5)	0.0227 (5)	0.0314 (5)	−0.0071 (4)	0.0049 (4)	0.0022 (4)
O2B	0.0322 (5)	0.0251 (5)	0.0344 (6)	−0.0063 (4)	0.0062 (4)	0.0114 (4)
O3B	0.0189 (5)	0.0264 (5)	0.0592 (8)	−0.0017 (4)	0.0099 (5)	−0.0102 (5)
O4B	0.0396 (6)	0.0262 (5)	0.0251 (5)	−0.0083 (4)	0.0055 (4)	−0.0046 (4)
N1B	0.0229 (6)	0.0323 (7)	0.0362 (7)	0.0093 (5)	−0.0073 (5)	−0.0167 (6)
C1B	0.0185 (5)	0.0213 (6)	0.0213 (6)	0.0016 (4)	0.0068 (4)	0.0015 (5)
C2B	0.0209 (5)	0.0167 (6)	0.0210 (6)	0.0027 (4)	0.0075 (4)	0.0014 (4)
C3B	0.0188 (5)	0.0154 (5)	0.0177 (5)	−0.0001 (4)	0.0050 (4)	0.0016 (4)
C4B	0.0195 (5)	0.0162 (5)	0.0200 (5)	0.0024 (4)	0.0032 (4)	0.0022 (4)
C5B	0.0240 (6)	0.0143 (5)	0.0183 (5)	0.0008 (4)	0.0033 (4)	0.0035 (4)
C6B	0.0219 (5)	0.0186 (6)	0.0170 (5)	−0.0030 (4)	0.0050 (4)	0.0030 (4)
C7B	0.0352 (8)	0.0188 (7)	0.0470 (9)	−0.0083 (6)	−0.0021 (7)	0.0091 (6)
C8B	0.0244 (6)	0.0281 (7)	0.0165 (5)	0.0037 (5)	0.0057 (4)	−0.0018 (5)
C9B	0.0233 (6)	0.0296 (7)	0.0169 (5)	0.0026 (5)	0.0059 (4)	0.0033 (5)
C10B	0.0173 (5)	0.0224 (6)	0.0203 (6)	−0.0005 (4)	0.0017 (4)	0.0013 (5)
C11B	0.0207 (5)	0.0251 (6)	0.0178 (5)	−0.0022 (5)	0.0043 (4)	−0.0040 (5)
C12B	0.0204 (5)	0.0248 (6)	0.0164 (5)	−0.0039 (5)	0.0051 (4)	−0.0034 (5)
C13B	0.0168 (5)	0.0226 (6)	0.0172 (5)	−0.0002 (4)	0.0026 (4)	−0.0027 (5)
C14B	0.0191 (5)	0.0229 (6)	0.0200 (6)	0.0007 (4)	0.0029 (4)	−0.0032 (5)
C15B	0.0217 (6)	0.0250 (6)	0.0229 (6)	0.0020 (5)	0.0023 (5)	−0.0057 (5)
C16B	0.0159 (5)	0.0244 (7)	0.0316 (7)	0.0030 (4)	−0.0031 (5)	−0.0114 (5)
C17B	0.0205 (6)	0.0213 (6)	0.0385 (8)	0.0000 (5)	0.0004 (5)	−0.0066 (6)
C18B	0.0226 (6)	0.0223 (7)	0.0585 (11)	0.0001 (5)	−0.0003 (7)	−0.0022 (7)
C19B	0.0253 (7)	0.0207 (7)	0.0758 (14)	0.0020 (5)	−0.0066 (8)	−0.0130 (8)
C20B	0.0277 (7)	0.0317 (8)	0.0610 (12)	0.0110 (6)	−0.0129 (7)	−0.0255 (8)
C21B	0.0395 (9)	0.0514 (11)	0.0283 (8)	0.0218 (8)	−0.0058 (6)	−0.0163 (7)

### Geometric parameters (Å, °)

**Table:**

Cl1A—C10A	1.7406 (14)	Cl1B—C10B	1.7378 (14)
S1A—O3A	1.4512 (11)	S1B—O4B	1.4526 (11)
S1A—O2A	1.4515 (11)	S1B—O2B	1.4542 (11)
S1A—O4A	1.4531 (11)	S1B—O3B	1.4558 (11)
S1A—C3A	1.7720 (13)	S1B—C3B	1.7742 (13)
O1A—C6A	1.3614 (16)	O1B—C6B	1.3680 (15)
O1A—C7A	1.434 (2)	O1B—C7B	1.4334 (19)
N1A—C20A	1.356 (2)	N1B—C20B	1.367 (2)
N1A—C16A	1.3744 (18)	N1B—C16B	1.3758 (18)
N1A—C21A	1.484 (2)	N1B—C21B	1.479 (2)
C1A—C2A	1.3821 (19)	C1B—C2B	1.3851 (18)
C1A—C6A	1.4011 (19)	C1B—C6B	1.3985 (18)
C1A—H1AA	0.9300	C1B—H1BA	0.9300
C2A—C3A	1.3998 (16)	C2B—C3B	1.3980 (17)
C2A—H2AA	0.9300	C2B—H2BA	0.9300
C3A—C4A	1.3908 (17)	C3B—C4B	1.3882 (17)
C4A—C5A	1.3918 (19)	C4B—C5B	1.3970 (18)
C4A—H4AA	0.9300	C4B—H4BA	0.9300
C5A—C6A	1.3941 (19)	C5B—C6B	1.3919 (18)
C5A—H5AA	0.9300	C5B—H5BA	0.9300
C7A—H7AA	0.9600	C7B—H7BA	0.9600
C7A—H7AB	0.9600	C7B—H7BB	0.9600
C7A—H7AC	0.9600	C7B—H7BC	0.9600
C8A—C9A	1.3853 (19)	C8B—C9B	1.384 (2)
C8A—C13A	1.4096 (18)	C8B—C13B	1.4053 (18)
C8A—H8AA	0.9300	C8B—H8BA	0.9300
C9A—C10A	1.3899 (18)	C9B—C10B	1.3867 (19)
C9A—H9AA	0.9300	C9B—H9BA	0.9300
C10A—C11A	1.3932 (18)	C10B—C11B	1.3940 (18)
C11A—C12A	1.383 (2)	C11B—C12B	1.3878 (19)
C11A—H11A	0.9300	C11B—H11B	0.9300
C12A—C13A	1.4052 (18)	C12B—C13B	1.4061 (18)
C12A—H12A	0.9300	C12B—H12B	0.9300
C13A—C14A	1.4642 (19)	C13B—C14B	1.4635 (19)
C14A—C15A	1.3429 (19)	C14B—C15B	1.3445 (19)
C14A—H14A	0.9300	C14B—H14B	0.9300
C15A—C16A	1.4556 (19)	C15B—C16B	1.457 (2)
C15A—H15A	0.9300	C15B—H15B	0.9300
C16A—C17A	1.393 (2)	C16B—C17B	1.393 (2)
C17A—C18A	1.385 (2)	C17B—C18B	1.385 (2)
C17A—H17A	0.9300	C17B—H17B	0.9300
C18A—C19A	1.389 (3)	C18B—C19B	1.389 (3)
C18A—H18A	0.9300	C18B—H18B	0.9300
C19A—C20A	1.368 (3)	C19B—C20B	1.365 (3)
C19A—H19A	0.9300	C19B—H19B	0.9300
C20A—H20A	0.9300	C20B—H20B	0.9300
C21A—H21A	0.9600	C21B—H21D	0.9600
C21A—H21B	0.9600	C21B—H21E	0.9600
C21A—H21C	0.9600	C21B—H21F	0.9600
			
O3A—S1A—O2A	113.28 (8)	O4B—S1B—O2B	112.80 (7)
O3A—S1A—O4A	112.81 (8)	O4B—S1B—O3B	113.00 (7)
O2A—S1A—O4A	112.52 (7)	O2B—S1B—O3B	113.16 (7)
O3A—S1A—C3A	105.18 (6)	O4B—S1B—C3B	106.07 (6)
O2A—S1A—C3A	105.55 (6)	O2B—S1B—C3B	105.50 (6)
O4A—S1A—C3A	106.70 (6)	O3B—S1B—C3B	105.45 (6)
C6A—O1A—C7A	116.49 (12)	C6B—O1B—C7B	116.86 (11)
C20A—N1A—C16A	121.34 (13)	C20B—N1B—C16B	120.48 (16)
C20A—N1A—C21A	117.29 (13)	C20B—N1B—C21B	118.41 (15)
C16A—N1A—C21A	121.37 (12)	C16B—N1B—C21B	121.11 (15)
C2A—C1A—C6A	119.76 (12)	C2B—C1B—C6B	119.71 (11)
C2A—C1A—H1AA	120.1	C2B—C1B—H1BA	120.1
C6A—C1A—H1AA	120.1	C6B—C1B—H1BA	120.1
C1A—C2A—C3A	120.53 (12)	C1B—C2B—C3B	120.24 (12)
C1A—C2A—H2AA	119.7	C1B—C2B—H2BA	119.9
C3A—C2A—H2AA	119.7	C3B—C2B—H2BA	119.9
C4A—C3A—C2A	119.14 (12)	C4B—C3B—C2B	119.53 (11)
C4A—C3A—S1A	121.17 (9)	C4B—C3B—S1B	121.50 (9)
C2A—C3A—S1A	119.69 (9)	C2B—C3B—S1B	118.96 (10)
C3A—C4A—C5A	121.08 (12)	C3B—C4B—C5B	120.94 (11)
C3A—C4A—H4AA	119.5	C3B—C4B—H4BA	119.5
C5A—C4A—H4AA	119.5	C5B—C4B—H4BA	119.5
C4A—C5A—C6A	119.14 (12)	C6B—C5B—C4B	118.84 (12)
C4A—C5A—H5AA	120.4	C6B—C5B—H5BA	120.6
C6A—C5A—H5AA	120.4	C4B—C5B—H5BA	120.6
O1A—C6A—C5A	124.16 (12)	O1B—C6B—C5B	124.06 (12)
O1A—C6A—C1A	115.51 (12)	O1B—C6B—C1B	115.23 (11)
C5A—C6A—C1A	120.32 (12)	C5B—C6B—C1B	120.70 (12)
O1A—C7A—H7AA	109.5	O1B—C7B—H7BA	109.5
O1A—C7A—H7AB	109.5	O1B—C7B—H7BB	109.5
H7AA—C7A—H7AB	109.5	H7BA—C7B—H7BB	109.5
O1A—C7A—H7AC	109.5	O1B—C7B—H7BC	109.5
H7AA—C7A—H7AC	109.5	H7BA—C7B—H7BC	109.5
H7AB—C7A—H7AC	109.5	H7BB—C7B—H7BC	109.5
C9A—C8A—C13A	120.99 (12)	C9B—C8B—C13B	121.06 (12)
C9A—C8A—H8AA	119.5	C9B—C8B—H8BA	119.5
C13A—C8A—H8AA	119.5	C13B—C8B—H8BA	119.5
C8A—C9A—C10A	119.10 (12)	C8B—C9B—C10B	119.36 (12)
C8A—C9A—H9AA	120.5	C8B—C9B—H9BA	120.3
C10A—C9A—H9AA	120.5	C10B—C9B—H9BA	120.3
C9A—C10A—C11A	121.55 (13)	C9B—C10B—C11B	121.42 (13)
C9A—C10A—Cl1A	119.29 (10)	C9B—C10B—Cl1B	118.32 (10)
C11A—C10A—Cl1A	119.15 (10)	C11B—C10B—Cl1B	120.26 (11)
C12A—C11A—C10A	118.76 (12)	C12B—C11B—C10B	118.64 (12)
C12A—C11A—H11A	120.6	C12B—C11B—H11B	120.7
C10A—C11A—H11A	120.6	C10B—C11B—H11B	120.7
C11A—C12A—C13A	121.45 (12)	C11B—C12B—C13B	121.42 (12)
C11A—C12A—H12A	119.3	C11B—C12B—H12B	119.3
C13A—C12A—H12A	119.3	C13B—C12B—H12B	119.3
C12A—C13A—C8A	118.14 (12)	C8B—C13B—C12B	118.10 (12)
C12A—C13A—C14A	118.49 (11)	C8B—C13B—C14B	122.27 (12)
C8A—C13A—C14A	123.37 (12)	C12B—C13B—C14B	119.63 (11)
C15A—C14A—C13A	124.53 (12)	C15B—C14B—C13B	123.55 (13)
C15A—C14A—H14A	117.7	C15B—C14B—H14B	118.2
C13A—C14A—H14A	117.7	C13B—C14B—H14B	118.2
C14A—C15A—C16A	123.89 (13)	C14B—C15B—C16B	123.70 (14)
C14A—C15A—H15A	118.1	C14B—C15B—H15B	118.1
C16A—C15A—H15A	118.1	C16B—C15B—H15B	118.1
N1A—C16A—C17A	118.05 (13)	N1B—C16B—C17B	118.35 (14)
N1A—C16A—C15A	118.24 (12)	N1B—C16B—C15B	118.13 (14)
C17A—C16A—C15A	123.72 (13)	C17B—C16B—C15B	123.52 (13)
C18A—C17A—C16A	120.94 (15)	C18B—C17B—C16B	121.37 (15)
C18A—C17A—H17A	119.5	C18B—C17B—H17B	119.3
C16A—C17A—H17A	119.5	C16B—C17B—H17B	119.3
C17A—C18A—C19A	119.08 (16)	C17B—C18B—C19B	118.62 (18)
C17A—C18A—H18A	120.5	C17B—C18B—H18B	120.7
C19A—C18A—H18A	120.5	C19B—C18B—H18B	120.7
C20A—C19A—C18A	119.48 (15)	C20B—C19B—C18B	119.79 (16)
C20A—C19A—H19A	120.3	C20B—C19B—H19B	120.1
C18A—C19A—H19A	120.3	C18B—C19B—H19B	120.1
N1A—C20A—C19A	121.09 (15)	C19B—C20B—N1B	121.37 (16)
N1A—C20A—H20A	119.5	C19B—C20B—H20B	119.3
C19A—C20A—H20A	119.5	N1B—C20B—H20B	119.3
N1A—C21A—H21A	109.5	N1B—C21B—H21D	109.5
N1A—C21A—H21B	109.5	N1B—C21B—H21E	109.5
H21A—C21A—H21B	109.5	H21D—C21B—H21E	109.5
N1A—C21A—H21C	109.5	N1B—C21B—H21F	109.5
H21A—C21A—H21C	109.5	H21D—C21B—H21F	109.5
H21B—C21A—H21C	109.5	H21E—C21B—H21F	109.5
			
C6A—C1A—C2A—C3A	−1.3 (2)	C6B—C1B—C2B—C3B	−1.37 (19)
C1A—C2A—C3A—C4A	0.37 (19)	C1B—C2B—C3B—C4B	1.88 (19)
C1A—C2A—C3A—S1A	−179.50 (10)	C1B—C2B—C3B—S1B	−177.39 (10)
O3A—S1A—C3A—C4A	3.32 (13)	O4B—S1B—C3B—C4B	−108.83 (11)
O2A—S1A—C3A—C4A	123.36 (12)	O2B—S1B—C3B—C4B	131.27 (11)
O4A—S1A—C3A—C4A	−116.73 (11)	O3B—S1B—C3B—C4B	11.27 (13)
O3A—S1A—C3A—C2A	−176.81 (11)	O4B—S1B—C3B—C2B	70.43 (12)
O2A—S1A—C3A—C2A	−56.77 (12)	O2B—S1B—C3B—C2B	−49.47 (12)
O4A—S1A—C3A—C2A	63.14 (12)	O3B—S1B—C3B—C2B	−169.48 (11)
C2A—C3A—C4A—C5A	0.64 (19)	C2B—C3B—C4B—C5B	−0.67 (19)
S1A—C3A—C4A—C5A	−179.48 (10)	S1B—C3B—C4B—C5B	178.59 (10)
C3A—C4A—C5A—C6A	−0.67 (19)	C3B—C4B—C5B—C6B	−1.04 (19)
C7A—O1A—C6A—C5A	−16.38 (19)	C7B—O1B—C6B—C5B	−4.32 (19)
C7A—O1A—C6A—C1A	163.36 (13)	C7B—O1B—C6B—C1B	174.76 (13)
C4A—C5A—C6A—O1A	179.42 (12)	C4B—C5B—C6B—O1B	−179.40 (12)
C4A—C5A—C6A—C1A	−0.31 (19)	C4B—C5B—C6B—C1B	1.57 (19)
C2A—C1A—C6A—O1A	−178.45 (12)	C2B—C1B—C6B—O1B	−179.49 (11)
C2A—C1A—C6A—C5A	1.3 (2)	C2B—C1B—C6B—C5B	−0.38 (19)
C13A—C8A—C9A—C10A	−0.7 (2)	C13B—C8B—C9B—C10B	−0.5 (2)
C8A—C9A—C10A—C11A	−0.2 (2)	C8B—C9B—C10B—C11B	1.0 (2)
C8A—C9A—C10A—Cl1A	178.29 (10)	C8B—C9B—C10B—Cl1B	−179.60 (11)
C9A—C10A—C11A—C12A	0.9 (2)	C9B—C10B—C11B—C12B	−0.4 (2)
Cl1A—C10A—C11A—C12A	−177.56 (10)	Cl1B—C10B—C11B—C12B	−179.82 (10)
C10A—C11A—C12A—C13A	−0.8 (2)	C10B—C11B—C12B—C13B	−0.6 (2)
C11A—C12A—C13A—C8A	−0.09 (19)	C9B—C8B—C13B—C12B	−0.5 (2)
C11A—C12A—C13A—C14A	179.97 (12)	C9B—C8B—C13B—C14B	179.93 (13)
C9A—C8A—C13A—C12A	0.84 (19)	C11B—C12B—C13B—C8B	1.10 (19)
C9A—C8A—C13A—C14A	−179.22 (13)	C11B—C12B—C13B—C14B	−179.34 (12)
C12A—C13A—C14A—C15A	172.88 (13)	C8B—C13B—C14B—C15B	−17.2 (2)
C8A—C13A—C14A—C15A	−7.1 (2)	C12B—C13B—C14B—C15B	163.30 (13)
C13A—C14A—C15A—C16A	178.86 (12)	C13B—C14B—C15B—C16B	178.33 (12)
C20A—N1A—C16A—C17A	1.17 (19)	C20B—N1B—C16B—C17B	0.2 (2)
C21A—N1A—C16A—C17A	−178.46 (12)	C21B—N1B—C16B—C17B	−179.88 (13)
C20A—N1A—C16A—C15A	−179.14 (12)	C20B—N1B—C16B—C15B	−179.62 (13)
C21A—N1A—C16A—C15A	1.22 (18)	C21B—N1B—C16B—C15B	0.34 (19)
C14A—C15A—C16A—N1A	−177.99 (13)	C14B—C15B—C16B—N1B	−166.95 (13)
C14A—C15A—C16A—C17A	1.7 (2)	C14B—C15B—C16B—C17B	13.3 (2)
N1A—C16A—C17A—C18A	0.2 (2)	N1B—C16B—C17B—C18B	1.1 (2)
C15A—C16A—C17A—C18A	−179.44 (13)	C15B—C16B—C17B—C18B	−179.12 (14)
C16A—C17A—C18A—C19A	−1.2 (2)	C16B—C17B—C18B—C19B	−1.1 (2)
C17A—C18A—C19A—C20A	0.7 (2)	C17B—C18B—C19B—C20B	−0.2 (2)
C16A—N1A—C20A—C19A	−1.6 (2)	C18B—C19B—C20B—N1B	1.5 (2)
C21A—N1A—C20A—C19A	178.03 (13)	C16B—N1B—C20B—C19B	−1.5 (2)
C18A—C19A—C20A—N1A	0.6 (2)	C21B—N1B—C20B—C19B	178.56 (15)

### Hydrogen-bond geometry (Å, °)

**Table:**

*D*—H···*A*	*D*—H	H···*A*	*D*···*A*	*D*—H···*A*
C1A—H1AA···O3B^i^	0.93	2.53	3.3040 (17)	141
C7A—H7AB···O3A^ii^	0.96	2.56	3.506 (2)	171
C1B—H1BA···O3A	0.93	2.31	3.2373 (17)	172
C12B—H12B···O2B^i^	0.93	2.47	3.2817 (17)	146
C7B—H7BA···O4A^ii^	0.96	2.57	3.518 (2)	171
C14B—H14B···O3B^i^	0.93	2.59	3.5204 (19)	176
C15A—H15A···O2A^iii^	0.93	2.47	3.2626 (19)	143
C17B—H17B···O3B^i^	0.93	2.58	3.474 (2)	161
C20A—H20A···O4A^iv^	0.93	2.50	3.159 (2)	128
C20B—H20B···O4B^v^	0.93	2.38	3.185 (2)	144
C21A—H21C···O2A^iii^	0.96	2.29	3.162 (2)	150
C21B—H21D···O2B^vi^	0.96	2.42	3.317 (2)	156
C9A—H9AA···Cg3^iii^	0.93	2.70	3.4423 (13)	137
C11A—H11A···Cg3	0.93	2.59	3.3482 (13)	139
C11B—H11B···Cg4^vii^	0.93	2.77	3.5457 (14)	142
C9B—H9BA···Cg4^vi^	0.93	2.62	3.3442 (13)	135
C21A—H21B···Cg2^iii^	0.96	2.82	3.6853 (18)	151
C21B—H21F···Cg1^viii^	0.96	2.82	3.7100 (19)	155

Symmetry codes: (i) *x*−1, *y*, *z*; (ii) −*x*+1, *y*−1/2, −*z*+1/2;  (iii) *x*, −*y*+3/2, *z*+1/2; (iv) −*x*+1, −*y*+1, −*z*+1; (v) *x*−1, −*y*+3/2, *z*−1/2; (vi) −*x*+1, −*y*+2, −*z*; (vii) −*x*+1, *y*+1/2, −*z*+1/2; (viii) *x*, −*y*+3/2, *z*−3/2.

## References

[bb1] Allen, F. H., Kennard, O., Watson, D. G., Brammer, L., Orpen, A. G. & Taylor, R. (1987). *J. Chem. Soc. Perkin Trans. 2*, pp. S1–19.

[bb2] Bruker (2005). *APEX2*, *SAINT* and *SADABS* Bruker AXS Inc., Madison, Wisconsin, USA.

[bb3] Chanawanno, K., Chantrapromma, S. & Fun, H.-K. (2008). *Acta Cryst.* E**64**, o1882–o1883. 10.1107/S1600536808027724 PMC2959251 21201095

[bb4] Chantrapromma, S., Chanawanno, K. & Fun, H.-K. (2009). *Acta Cryst.* E**65**, o1144–o1145. 10.1107/S1600536809014974 PMC2977813 21583950

[bb5] Chantrapromma, S., Rodwatcharapiban, P. & Fun, H.-K. (2006). *Acta Cryst.* E**62**, o5689–o5691.

[bb6] Chantrapromma, S., Ruanwas, P., Fun, H.-K. & Patil, P. S. (2006). *Acta Cryst.* E**62**, o5494–o5496.

[bb7] Cheng, L. T., Tam, W., Marder, S. R., Stiegman, A. E., Rikken, G. & Spangler, C. W. (1991). *J. Phys. Chem* **95**, 10643–10652.

[bb8] Cheng, L. T., Tam, W., Stevenson, S. H., Meredith, G. R., Rikken, G. & Marder, S. R. (1991). *J. Phys. Chem* **95**, 10631–10643.

[bb9] Cosier, J. & Glazer, A. M. (1986). *J. Appl. Cryst.* **19**, 105–107.

[bb10] Sheldrick, G. M. (2008). *Acta Cryst.* A**64**, 112–122. 10.1107/S0108767307043930 18156677

[bb11] Spek, A. L. (2009). *Acta Cryst.* D**65**, 148–155. 10.1107/S090744490804362X PMC2631630 19171970

